# MM120 (Lysergide) May Enhance Neuroplasticity by Post-Dose Upregulation of TrkB

**DOI:** 10.1192/j.eurpsy.2026.11607

**Published:** 2026-07-31

**Authors:** G. Smagin, J. Tripp

**Affiliations:** Non-Clinical, MindMed, New York, United States

## Abstract

**Introduction:**

MM120 (lysergide D-tartrate, a formulation of LSD) is under development as a potential treatment for generalized anxiety and major depressive disorders. How psychedelic drugs interact at the TrkB receptor, a key target of neuroplasticity, is not reported.

**Objectives:**

The study evaluated binding and functional activity of known serotonergic psychedelic compounds and SRI fluoxetine at the TrkB receptor.

**Methods:**

Activation of TrkB by brain-derived neurotrophic factor (BDNF) was assessed by measuring inositol monophosphate 1 (IP1) accumulation. NIH/3T3 cells stably expressing human TrkB were used. BDNF was added, followed by incubation with Anti-IP1-Cryptate and IP1-d2. IP1 formation was determined by Homogeneous Time-Resolved Fluorescence (HTRF). We tested LSD, psilocin, N,N-dimethyltryptamine (DMT), mescaline, 2,5-dimethoxy-4-iodoamphetamine (DOI),N-2-methoxybenzyl- phenethylamine (25B-NBOMe), 4-bromo-2,5-dimethoxyphenethylamine (2C-B), methylenedioxyamphetamine (MDA), and the selective serotonin reuptake inhibitor fluoxetine. Activation potencies (EC_50_) were derived from the concentration-response curves using nonlinear regression.

**Results:**

BDNF was highly potent at the TrkB receptor, activating it in the sub-picomolar range (EC_50_ 0.2 pM). LSD, 25B-NBOMe, and fluoxetine activated TrkB (EC_50_ of 811, 26370, and 6040 pM respectively) with low maximal efficacies (40, 57, and 60% respectively). All remaining drugs did not activate the TrkB at concentrations <1 mM. Cotreatment with BDNF and a fixed concentration of LSD increased the BDNF-induced TrkB activation potency (EC_50_ 0.06 pM) and reduced the activation efficacy to 60%. Other drugs reduced the activation potency, as well as the maximal receptor activation.

**Image 1:**
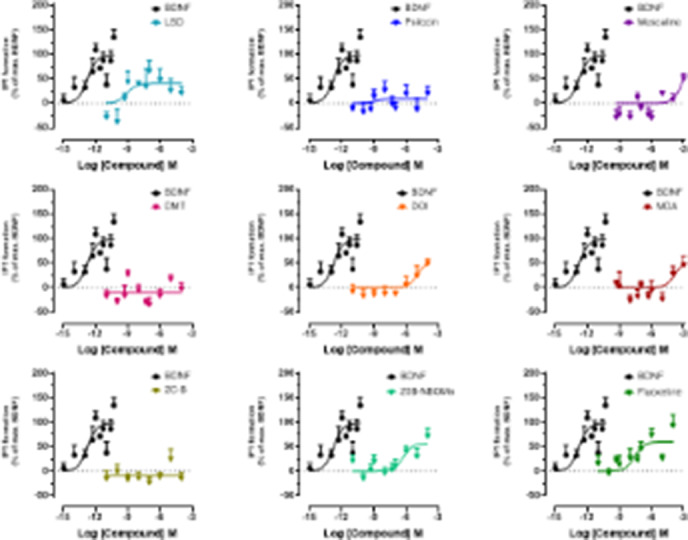
Image 1: Long description.

**Conclusions:**

LSD, 25B-NBOMe, and fluoxetine activated TrkB at pharmacologically relevant concentrations as partial agonists, while psilocin, DMT, DOI, MDA, and 2C-B did not. Induced TrkB upregulation may underly and provide evidence for the neuroplastic effects of LSD.

**Disclosure of Interest:**

G. Smagin Shareolder of: 100%, Employee of: 100%, J. Tripp Shareolder of: 100%, Employee of: 100%

## Long descriptions

### Image 1: Long description

A multi-panel figure containing 9 individual line graphs arranged in a 3 by 3 grid. All graphs share identical axes. The y-axis represents I P 1 formation as a percentage of maximum B D N F, ranging from negative 50 to 200. The x-axis represents Log open bracket Compound close bracket M, ranging from negative 15 to negative 3.

In every panel, a black curve representing B D N F shows a sigmoidal increase starting at negative 15 and peaking around negative 10. Each panel compares this to a different compound:

* Top-left: L S D (light blue) shows a low-amplitude bell-shaped curve peaking at negative 6.

* Top-center: Psilocin (blue) shows a flat response near zero.

* Top-right: Mescaline (purple) shows a flat response until negative 4, where it begins to rise.

* Middle-left: D M T (pink) shows a flat response near zero.

* Middle-center: D O I (orange) shows a flat response until negative 6, followed by a slight increase.

* Middle-right: M D A (red) shows a flat response near zero.

* Bottom-left: 2 C dash B (olive) shows a flat response near zero.

* Bottom-center: 25 I dash N B O Me (teal) shows a sigmoidal increase starting at negative 8.

* Bottom-right: Fluoxetine (green) shows a sigmoidal increase starting at negative 9, reaching a plateau around 50 percent.

Navigate back to Image 1:.

